# Multidimensional Evaluation of the Process of Constructing Age-Friendly Communities Among Different Aged Community Residents in Beijing, China: Cross-Sectional Questionnaire Study

**DOI:** 10.2196/66248

**Published:** 2025-03-21

**Authors:** Yingchun Peng, Zhiying Zhang, Ruyi Zhang, Yiyao Zhang, Runying Wang, Jiaying Zhang, Shaoqi Zhai, Qilin Jin, Jiaojiao Zhou, Jingjing Chen

**Affiliations:** 1 School of Medical Humanities, Capital Medical University, No.10, Xitoutiao, You An Men Wai, Beijing, 100069, China, 86 13241902279; 2 Outpatient Office, Beijing Luhe Hospital, Capital Medical University, Beijing, China; 3 Ethics Committee Office, Beijing Ditan Hospital, Capital Medical University, Beijing, China; 4 Cardiac Surgery Department, People’s Hospital of Beijing Daxing District, Beijing, China; 5 Medical Department, Fengtai District Xiluoyuan Community Health Service Center, Beijing, China; 6 Huairou District Liulimiao Community Health Service Center, Beijing, China

**Keywords:** age-friendly, positive living experience, active aging, healthy aging, community residents, older adults, age-friendly communities, multiple stakeholders, evaluating age-friendliness, urban and suburban areas

## Abstract

**Background:**

The World Health Organization (WHO) has made significant efforts to promote age-friendly community initiatives (AFCI) to address the challenges of population aging. Previous studies have discussed the construction of age-friendly communities (AFC) in urban cities, evaluating AFCs often rooted in the WHO’s Checklist and focused on a single group, namely older adults, overlooking the role of other age groups in community development.

**Objective:**

This study aims to evaluate AFCs from multidimensional aspects, particularly the positive living experiences of older adults, summarize the deficiencies in both hardware and software aspects in the process of constructing AFCs in China, and provide some recommendations to promote AFCIs worldwide.

**Methods:**

Using a multistage sampling strategy, 470 community residents from urban and suburban areas participated in this study. A self-designed questionnaire was designed to use a standardized method to evaluate older adults’ living experiences across five dimensions, including the degree of age-friendliness in the community, social support, sense of gain, sense of happiness, and sense of security. Respondents rated each dimension on a 10-point scale. This study defined community residents into 3 groups: residents younger than 45 years(Group 1: youth), those aged 45-59 years (Group 2: middle-aged), and those aged ≥60 years (Group 3: old-age).

**Results:**

In this study, 382 (81.3%) community residents were unaware of the relevant concepts of AFCs. Most participants highlighted the importance of community support and health services, followed by respect and social inclusion, and outdoor spaces and buildings. The findings showed that the highest-rated dimension was the sense of security. The mean scores for the degree of the sense of security in urban and suburban areas were 7.88 (SD 1.776) and 7.73 (SD 1.853), respectively. For Group 2, the mean scores were 7.60 (SD 2.070) and 8.03 (SD 1.662), while Group 3 had mean scores of 7.34 (SD 2.004) and 7.91 (SD 1.940). The lowest-rated dimension was social support; the mean scores for Group 1 for the degree of social support in urban and suburban areas were 7.63 (SD 1.835) and 7.48 (SD 1.918), respectively. For Group 2, the mean scores were 6.94 (SD 2.087) and 7.36 (SD 2.228), while those for Group 3 were 6.37 (SD 2.299) and 6.84 (SD 2.062). Further, there were significant differences in the scores of residents among different age groups in urban areas regarding age-friendliness (*P*<.001), social support, (*P*<.001), and sense of gain (*P*=.01).

**Conclusions:**

China is in the early stages of developing AFCs. We further highlight the importance of continued research on the collaboration and participation among multiple stakeholders. These outcomes have a direct and positive impact on the well‐being of older adults.

## Introduction

### The History and Imperative of Implementing Age-Friendly Communities

Population aging is poised to become one of the most significant problems in the 21st century, impeding the development of many countries globally. Data from *World Populations Prospects* [1] predicated that the number of persons aged ≥65 years in countries and areas with populations that have already peaked is projected to reach 409 million by 2027, surpassing the number of children under 18. The number of persons aged ≥80 years is increasing at an even faster rate and is likely to triple, from 85 million in 2024 to approximately 267 million by 2100. With the increasing trend of population aging, health issues in older adults have become a major concern for scholars and policymakers worldwide. As people age, many experience a myriad of changes impacting their health and functional ability to manage independent living in their homes and communities [2]. Meanwhile, age-related changes such as physiological and cognitive decline may lead to negative health outcomes such as frailty, mobility limitations, and disability. Moreover, many social and physical environmental factors also affect the health status and quality of life of older adults [3,4]. Therefore, finding accessible and affordable measures to help older adults live longer and healthier will be crucial.

To address the challenges of population aging and achieve the goal of healthy aging, the World Health Organization(WHO) has made significant efforts to promote age-friendly community initiatives (AFCI). The concept of age-friendly communities (AFCs) originated from the Ecology Theory of Aging in 1973 [5,6], which indicated that aging was a dynamic process at both the individual and community levels, revealing that a positive surrounding environment can promote well-being among older adults. In 2000, the American Association of Retired Persons first defined AFCs as communities with affordable housing, adequate infrastructure and services, and diverse options of transportation. Based on the report *A Guide: Global Age-Friendly Cities* [7], the AFCs incorporated three aspects and eight key domains. Three aspects included service support, space environment, social and humanistic environment [8], and the eight key domains consisted of outdoor spaces and buildings, transportation, housing, social participation, respect and social inclusion, civic participation and employment, communication and information, and community and health services [9,10]. In the current conceptualization of AFCs, the importance of social environment and physical environment are placed equally as the determinants of health and older adults are considered to be effective social resources involved in community construction [11]. The AFCs are designed to account for the wide diversity among older people and promote their autonomy, inclusion, and contributions in all areas of community life [12].

Meanwhile, the implementation of aging-in-place policies highlights the imperative for AFCIs [13]. Supporting older adults to continue living safely and comfortably within their communities is a shared vision of many governments and international organizations. Due to their continuous efforts, the WHO Global Network for AFCIs currently includes 1705 cities and communities across 60 countries, covering over 330 million people worldwide. However, the lack of measurability of the actual age-friendliness of AFCs has been a major weakness in moving AFCIs forward. As age-friendliness is a complex, dynamic, multidimensional, and highly context-dependent concept, it does not easily lend itself to standardization of measurement [14-16].

### Evaluating the Age-Friendliness of Communities

While constructing AFCs, it is crucial to evaluate the age-friendliness of communities [17]. The previous studies have mainly focused on measuring the age-friendliness of a city or community in which older adults live, based on the WHO’s Checklist [18]. Jo-Ying Huang et al [19] developed indicators of age-friendliness for communities in Taiwan province that conform to international standards by referring to the WHO’s Checklist and Taiwan’s existing indicators. Yu, Wong, and Woo [20] examined the relationships between perceptions of neighborhood environment, sense of community, and self-rated health by relying on the WHO Checklist. Wood et al [21] conducted a systematic scoping review of 23 articles using citizen science or participatory approaches. An inductive and deductive thematic analysis was performed to identify local urban barriers and facilitators and map them against the WHO’s Checklist. Kim et al [22] used data from the American Association of Retired Persons AFC surveys to evaluate the reliability and concurrent validity of AFC assessment tools. They found that higher levels of perceived availability of community and health services were associated with worse health outcomes.

Additionally, the Age Friendly Cities and Communities Questionnaire (AFCCQ) is designed by Dikken for measuring age-friendliness. Compared with WHO’s Checklist, the Age Friendly Cities and Communities Questionnaire only has 23 items, covers eight domains of the WHO’s AFC model, and has an additional ninth domain of financial situation [23]. In addition to the above-mentioned measurement tools of age-friendliness, sets of priority indicators, frameworks, and conceptual surveys are proposed by other studies. Jiravanichkul et al [24] identified minimum standard indicators and explored the development of a well-being environment and AFC assessment criteria by using the analytic hierarchy process. Ide et al [17] developed a community-level AFC indicator based on the WHO AFC guidelines by incorporating dementia-friendly elements and tested its validity and reliability.

### Beijing as a Case Example

Beijing is the capital of China, and a municipality directly governed by the Central Government. It is famous as China’s political, cultural, international communication, and science and technology innovation center. Beijing currently has entered a moderately aging society. By the end of 2023, the permanent resident population aged 60 years and above was 4.948 million, accounting for 22.6% of the total permanent resident population. The resident population aged 65 years and above was 3.469 million, accounting for 15.9% of the total resident population [25]. To enhance older adults’ sense of gain, happiness, and security in communities, the National Health Commission (National Office for the Elderly, 2020) launched an initiative to construct AFCs across the country. By the end of 2023, a total of 93 communities in Beijing had been named “ National Model AFCs,” which were at the forefront of AFC development nationwide.

### Focus of the Current Study

Although many scholars have attempted to evaluate the age-friendliness of communities in various ways, there is limited research emphasizing the positive aspects of health, such as the sense of gain, happiness, and security that older adults experience in their communities. Additionally, most studies have focused on the construction of AFCs in urban cities [26,27], and evaluating the age-friendliness of communities was only based on assessments from older adults [22,28], which overlooked the perspectives of other community residents. Therefore, the current evaluation of constructing AFCs may lack objectivity and accuracy.

This study aims to explore the cognition of AFCs among the different aged communities’ residents in Beijing, evaluate AFCs from multidimensional aspects, especially the positive living experience of older adults, summarize the deficiencies in hardware and software infrastructure in the process of constructing AFCs in Beijing, and provide a reference for promoting AFCIs across the world.

## Methods

### Study Design

This is a cross-sectional study, designed to understand the awareness and perception of AFCs among different aged community residents, explore deficiencies in the process of constructing AFCs, and propose recommendations for improvement.

### Study Setting and Sample

Considering the level of economic development and construction status of AFCs among 16 districts in Beijing, a cross-sectional quantitative survey was conducted on community residents between urban and suburban areas. This study adopted a multistage sampling strategy [29,30]. In the first stage, Xicheng District, Fengtai District, Daxing District, and Huairou District were selected based on the functional orientation of the 16 districts in Beijing and the current situation of constructing AFCs. To implement urban strategic positioning, promoting sustainable development, and considering the practical needs of the historical pattern of the ancient capital, Beijing issued the Beijing Urban Master Plan in 2017. According to the Beijing Urban Master Plan, there are six central urban areas (including Dongcheng District, Xicheng District, Chaoyang District, Haidian District, Fengtai District, and Shijingshan District), five new towns in plain areas (including Shunyi District, Daxing District, Yizhuang District, Fangshan District, and Changping District), and six ecological conservation areas (including Mentougou District, Pinggu District, Huairou District, Miyun District, and Yanqing District). For this study, plain new towns and ecological conservation areas were unified as suburban areas, while the central urban areas were unified as urban areas. Among the 16 districts, Xicheng District, Fengtai District, Daxing District, and Huairou Districts ranked second, third, fifth, and eighth, respectively. In the second stage, 3‐5 communities were randomly picked from each district, amounting to 15 communities in the list. In the last stage, trained investigators visited each sampling community to randomly invite community residents to participate in our investigation. Potential participants were recruited via the invitation of the investigators, and we sought to balance the sample based on population characteristics such as gender, age, education, and region. To better understand the construction of AFCs, our goal was to recruit residents who lived in the communities and who could freely and voluntarily express their insights.

### Measures

A self-designed questionnaire was developed to use a standardized method for assessing the residents’ living experience and their community’s age-friendliness in Beijing. It was designed in consultation with a group of AFC experts [31].

To explore the cognition of community residents regarding the concept of AFCs, the questionnaire included questions such as whether they had heard of or learned about AFCs. Based on our literature review and WHO guidelines, we asked residents to choose the most important domain among the eight domains (outdoor spaces and buildings, transportation, housing, social participation, respect and social inclusion, civic participation and employment, communication and information, and community and health services) in the process of constructing AFCs.

The positive living experience of older adults that was obtained within their community was measured across five dimensions: (1) the degree of age-friendliness in the community, (2) the level of social support for older adults, (3) the sense of gain in community, (4) the sense of happiness in the community, and (5) the sense of security in the community. Respondents rated each dimension on a 10-point scale (0‐1: does not exist, 2‐3: poor, 4‐5: fair, 6‐7: good, 8‐9: very good, 10: excellent).

To understand the shortcomings in the current process of constructing AFCs, the deficiencies were categorized into hardware and software aspects. Hardware aspects included increasing the green spaces and beautiful buildings, building more nursing institutions and medical institutions for older adults, building more leisure and entertainment places, installing more age-friendly facilities for older adults, and expanding traffic roads, among others. Software aspects consisted of conducting more abundant recreational activities, creating an age-friendly atmosphere, providing personalized elderly care services and community and neighborhood support, providing community volunteer services, and more personalized medical services. Participants were asked to choose the items that required urgent improvement in their communities.

### Data Analysis

Based on the latest WHO age definition criteria [32], this study defined community residents into three age groups: (1) Group 1 as youth (younger than 45 years) (2) Group 2 as middle-aged adults (45-59 years), and (3) Group 3 as old-aged adults (above 60 years).

All the gleaned data were recorded into EpiData software (version 3.1; EpiData Association) and analyzed by SPSS software (version 21.0; IBM Corp). The mean and standard deviation were used to describe continuous data, while the categorical data were presented by composition ratio, frequency distributions, and parity arrangement. Frequency and rank analyses were used to summarize the quantitative data of community residents including demographic characteristics such as gender, age, region, and education level. The rank-sum test was used to analyze the most important domains of AFCs and deficiencies in the construction of the AFCs. One-way ANOVA was used to explore the five dimensions of the community living experiences of older adults.

### Ethical Considerations

The study was approved by the Medical Ethics Committee of Capital Medical University, Beijing, China (Reference number Z2023SY048). All participants provided informed consent for the collection, handling, and storage of their personal and health data. All procedures were performed in accordance with relevant guidelines and regulations. All participants joined the study voluntarily, and no compensation was provided to them. All data were kept confidential, deidentified, and anonymous.

## Results

A total of 477 residents voluntarily agreed to participate in our survey. We collected 470 valid questionnaires were collected, resulting in a validity rate of 98.53%. Table 1 presents the demographic characteristics of community residents. Among the 470 community residents in our survey, the majority were female (n=339, 72.1%). Approximately 44.5% (n=209) of the participants were younger than 44 years, and the average age of the 470 community residents was 46.74 (SD 18.63) years. A total of 259 (55.1%) community residents were from urban areas. Additionally, 113 participants (24%) had lived in their current community for more than 30 years and 178 (37.9%) participants had obtained a bachelor’s degree.

**Table 1.** Demographic characteristics of community residents.

Items	Surveys (N=470), n (%)
Gender	
Male	131 (27.9）
Female	339 (72.1）
Age (years）	
≤44	209 (44.5）
45-59	132 (28.1）
≥60	129 (27.4）
Regions	
Urban areas	259 (55.1）
Suburban areas	211 (44.9）
Length of residence (years）	
≤10	157 (33.4）
11-30	200 (42.6）
31-50	53 (11.3）
51-70	42 (8.9）
≥71	18 (3.8）
Education	
Junior high school or below	129 (27.4）
High school or Junior college	99 (21.1）
Bachelor’s degree	178 (37.9）
Master’s degree or above	64 (13.6）

Table 2 shows community residents’ cognition of AFCs. Almost 81.3% of the community residents were not aware of the concept of AFCs; only 88 (18.7%) community residents had heard of AFCs.

**Table 2.** Community residents’ cognition of the Age-Friendly Communities.

Items	Results
Community residents’ cognition of the concept of the Age-Friendly Communities (N=470), Surveys, n (%)	
Know	88 (18.7)
Not know	382 (81.3)
The most important domain of the Age-Friendly Communities from the perspective of community residents, total points^a^ (rank)	
Outdoor spaces and buildings	453 (3)
Transportation	260 (6)
Housing	293 (5)
Social participation	364 (4)
Respect and social inclusion	559 (2)
Job opportunities and civic participation	84 (8)
Communication and information	135 (7)
Community support and health services	666 (1)

a Note: total points: number of people selected for the first important domain ×3 + number of people selected for the second important domain ×2 +number of people selected for the third important domain ×1.

With regard to the eight important domains of creating an AFC, community support and health services were the most important domains from the perspective of residents when they lived in the community, followed by respect and social inclusion, and outdoor spaces and buildings. Job opportunities and civic participation were considered relatively less important for residents when they lived in the community.

Table 3 displays an evaluation of the living experiences of older adults, categorized by different aged community residents. Among the five dimensions of older adults’ living experience, the scores of the sense of security in the community were relatively high, whereas the scores for the degree of social support for older adults were relatively low. There were significant differences in the scores of residents between different age groups in urban areas in terms of age-friendliness (*P*<.001), social support (*P*<.001), and sense of gain (*P*=.01), whereas no significant differences were found in terms of happiness and security. In the evaluation of age-friendliness, there were significant differences between young and middle-aged residents, and between young residents and elderly residents. In the evaluation of the degree of social support, there were significant differences between young residents and middle-aged residents, and between young residents and elderly residents. Additionally, the sense of gain differed significantly between young residents and elderly residents.

**Table 3.** Evaluation of living experience of older adults from the perspectives of different aged community residents.

Regions, items, and groups	Scores^a^ , mean (SD）	*F* test (*df*)	*P *value
Urban areas			
The degree of age-friendliness		9.687（2, 256）	<.001
Group 1（age <45 years)	7.82 (1.807)^a^		
Group 2 (age 45-60 years)	7.34 (1.689)^a^		
Group 3 （age ≥60 years)	6.58 (2.208)^b^		
The degree of social support		8.914（2, 256）	<.001
Group 1（age <45 years)	7.63 (1.835)^a^		
Group 2 (age 45-60 years)	6.94 (2.087)^b^		
Group 3 (age ≥60 years)	6.37 (2.299)^b^		
The sense of gain in community		4.445（2, 256）	.01
Group 1 (age <45 years)	7.58 (1.693)^a^		
Group 2 (age 45-60 years)	7.10 (1.995)^ab^		
Group 3 (age ≥60 years)	6.79 (1.880)^b^		
The sense of happiness in community		2.741（2, 256）	.07
Group 1 (age <45 years)	7.72 (1.777)		
Group 2 (age 45-60 years)	7.27 (1.933)		
Group 3 (age ≥60 years)	7.09 (2.079)		
The sense of security in community		1.792（2, 256）	.17
Group 1 (age <45 years)	7.88 (1.776)		
Group 2 (age 45-60 years)	7.60 (2.070)		
Group 3 (age ≥60 years)	7.34 (2.004)		
Suburban areas			
The degree of age-friendliness		1.290（2, 208）	.28
Group 1 (age <45 years)	7.61 (1.914)		
Group 2 (age 45-60 years)	7.44 (2.232)		
Group 3 (age ≥60 years)	7.05 (1.910)		
The degree of social support		1.732（2, 208）	.18
Group 1 (age <45 years)	7.48 (1.918)		
Group 2 (age 45-60 years)	7.36 (2.228)		
Group 3 (age ≥60 years)	6.84 (2.062)		
The sense of gain in community		0.138（2, 208）	.87
Group 1 (age <45 years)	7.29 (1.923)		
Group 2 (age 45-60 years)	7.41 (2.045)		
Group 3 (age ≥60 years)	7.21 (2.145)		
The sense of happiness in community		0.519( 2, 208）	.60
Group 1 (age <45 years)	7.53 (1.719)		
Group 2 (age 45-60 years)	7.51 (2.081)		
Group 3 (age ≥60 years)	7.82 (1.724)		
The sense of security in community		0.561（2, 208）	.57
Group 1 (age <45 years)	7.73 (1.853)		
Group 2 (age 45-60 years)	8.03 (1.662)		
Group 3 (age ≥60 years)	7.91 (1.940)		

a Different letters within the same row indicate statistically significant differences between groups based on the Student-Newman-Keuls (SNK) multiple comparisons (*P*<.05). Groups sharing the same letter do not differ significantly.

Table 4 presents the shortcomings in the current process of constructing AFCs. Regarding the aspects of community hardware facilities, most residents expressed the need for building more nursing institutions for older adults, followed by building additional medical institutions and installing more suitable age-friendly facilities. Expanding traffic roads was an aspect that concerned fewer people. Regarding the community software facilities, creating an age-friendly atmosphere was most commonly reported by many community residents, followed by providing personalized elderly care services, more personalized medical services, and more recreational activities.

**Table 4.** The shortcomings in the current process of constructing age-friendly communities.

Items	Frequency	Rank
The aspects of community hardware facilities that should be improved		
Increase the green area and beautiful buildings	217	5
Build more nursing institutions for older adults	319	1
Build more medical institutions for older adults	257	2
Build more leisure and entertainment places	221	4
Install more suitable facilities for older adults	252	3
Expand traffic roads	78	6
Others	11	7
The aspects of community software facilities that should be improved		
Hold more abundant recreational activities	207	4
Create an age-friendly atmosphere	323	1
Provide personalized elderly care services	317	2
Provide community and neighborhood support	117	6
Provide community volunteer services	170	5
Provide more intimate medical services	241	3
Others	13	7

## Discussion

### Principal Findings

This study is the first to comprehensively evaluate AFCs from multidimensional aspects in China. It particularly examines the positive living experiences that residents derive from their communities, compares the cognition and perception of AFCs among different aged community residents in urban and suburban areas, and explores the deficiencies in the process of constructing AFCs from both hardware and software perspectives. The community residents’ suggestions for improvement for constructing AFCs reflect the needs and interests of older adults and other age groups, providing a direction for constructing AFCs in the future.

#### Increasing Age-Friendly Awareness and Sense of Belonging Among Community Residents

China has only recently begun promoting AFCIs; compared to other developed countries, there remains significant progress to be made. Consequently, the public is not aware of the concepts of AFCs, which is consistent with our findings as only 88 (18.7%) residents were aware of AFCs. Recognizing the age-friendliness of communities is the premise to engage residents in the construction of AFCs. Relevant studies show that perceived age-friendliness of the community is positively associated with a sense of belonging to the community [33,34]. However, a gap exists between family belonging and community belonging. Given the changes in China’s economic and social structures, the sense of community belonging has been weakened [35], which may have led to a lack of initiative and consciousness among residents in the process of creating AFCs. Therefore, it is vital to improve the consciousness of age-friendliness and strengthen the sense of belonging among community residents in the process of constructing AFCs.

#### Establishing an Age-Friendly Atmosphere and Improving Communities’ Software Facilities

Previous studies have shown that constructing AFCs mainly focuses on eight domains, based on the WHO guideline [14,36]; however, little is known about which domains are considered most important for community residents. In our study, most participants highlighted the importance of community support and health services; they wish to build more medical institutions and provide more personalized medical services in their communities, which reflects the high demand for medical services by community residents in Beijing. However, a major challenge faced by both high-income and low-income countries today is inequitable access to health care resources [37-39]. For example, in suburban or medically underprivileged communities, residents may often be unable to access desired health care services. Telemedicine in China has been improving the dissemination of high-quality health care resources from urban cities to remote suburban areas, providing services such as teleconsultations and specialist diagnoses [37]. Meanwhile, respect and social inclusion are also key components for promoting AFCIs. Many community residents identified that creating an age-friendly atmosphere was the most important step in the process of AFCs to improve the aspects of community software facilities, which is consistent with other studies. Lui et al [13] noted that a supportive environment characterized by positive relations, engagement, and inclusion is a core prerequisite for aging well. Rémillard et al [40] have explored eleven case studies of AFCs worldwide and reported that the need to shift perception, change mindsets, and promote a more positive view of aging were identified as key priorities. Respect and promoting social participation of older adults is also seen as a way to challenge ageism. In addition, the range of recreational activities and social support for community residents within their communities should be increased. Some studies found a positive association between employment, social participation, and healthy aging [41]. These observations could be due to the potential of these activities to enhance individuals’ social status, promote psychological well-being, and foster a sense of dignity.

#### Improving the Living Environment and Strengthening Communities’ Hardware Facilities

**Figure 1.**
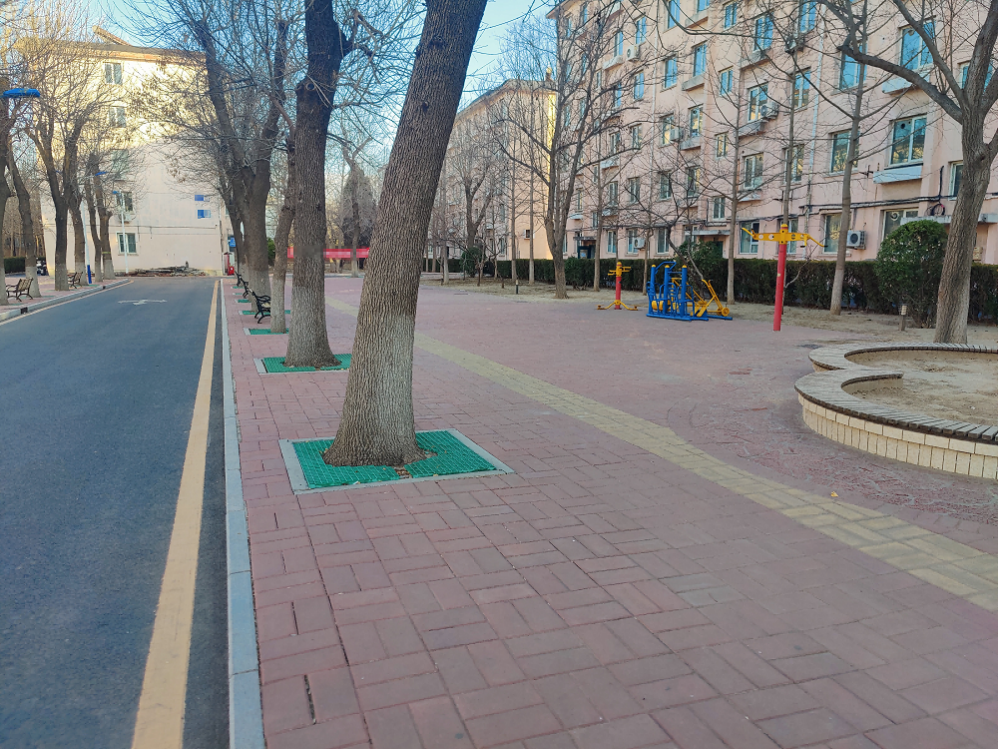
Overview of hardware facilities in a surveyed community in the Daxing District, showing limited public space, scarcity of age-appropriate fitness equipment, and no elevators.

Our results indicate that many community residents wish to improve the physical infrastructure of their communities; outdoor spaces and buildings were the third most important domain of AFCs. This finding is in line with the reality of communities’ infrastructure in China. Most older communities in Beijing lack elevators, which causes major inconvenience for older adults when going out. In addition, there is limited public space for residents to participate in activities and exercise. A common phenomenon in many old communities is that public spaces meant for residents’ activities are occupied by parked cars. As shown in Figure 1, limited public space, scarcity of age-appropriate fitness equipment, and the absence of elevators are key physical environment factors that may hinder older adults from participating in community activities.

#### Actively Participating in Community Activities to Enhance Community Residents’ Happiness

In the process of constructing AFCs, the notion of healthy aging should be considered the final goal. Older adults in good health can continue to be vital societal resources, experience greater job satisfaction and independence in their lives, and engage more actively as community residents. This study aimed to evaluate the positive living experiences that older adults obtained in their communities. Our findings show that among the five dimensions assessed, the sense of security scored the highest, while social support scored the lowest, particularly among urban older adults. Social support is a well-recognized social determinant of health, and it is obvious that obtaining social support from others is crucial throughout life, including old age [42]. Most older adults consistently prefer aging in place, which requires a high level of social support in the process of constructing AFCs [11]. Therefore, improving the degree of social support for older adults should be a key focus while constructing AFCs in the future. Further, there were significant differences in the evaluation of older adults’ living experiences among different aged community residents in urban areas. Young residents rated the degree of social support, age-friendliness, sense of gain, and happiness significantly higher than older adults’ self-evaluations. This disparity may stem from younger residents having greater physical capacity and healthier habits, which enables them to afford expenses related to transportation, health care, and retirement [41]. Consequently, they have more energy and capacity to actively engage in paid work, social participation, and environmental preservation, thereby fostering their sense of happiness and sense of gain. Besides, elderly residents in suburban areas have the highest scores for the sense of happiness, which suggests that older adults in suburban communities experience higher levels of happiness compared to their urban elderly residents. These findings may be attributed to both the physical environment (eg, accessible public facilities) and the social environment (eg, active engagement in volunteer activities), providing sufficient support to older adults in suburban areas [41]. Suburban seniors typically lived in the rural society for a long time. At the heart of rural society lies a deep-rooted connection to the land. Life in these communities is often dictated by the cycles of nature, with agricultural activities and environmental stewardship forming the backbone of their livelihoods. Therefore, compared with urban elderly residents, suburban seniors may engage in agricultural activities within their capacity, establish trust-based relationships with neighbors, and be more likely to participate in communities’ activities.

#### Participation of Multiple Stakeholders in the Promotion of Constructing AFCs

**Figure 2.**
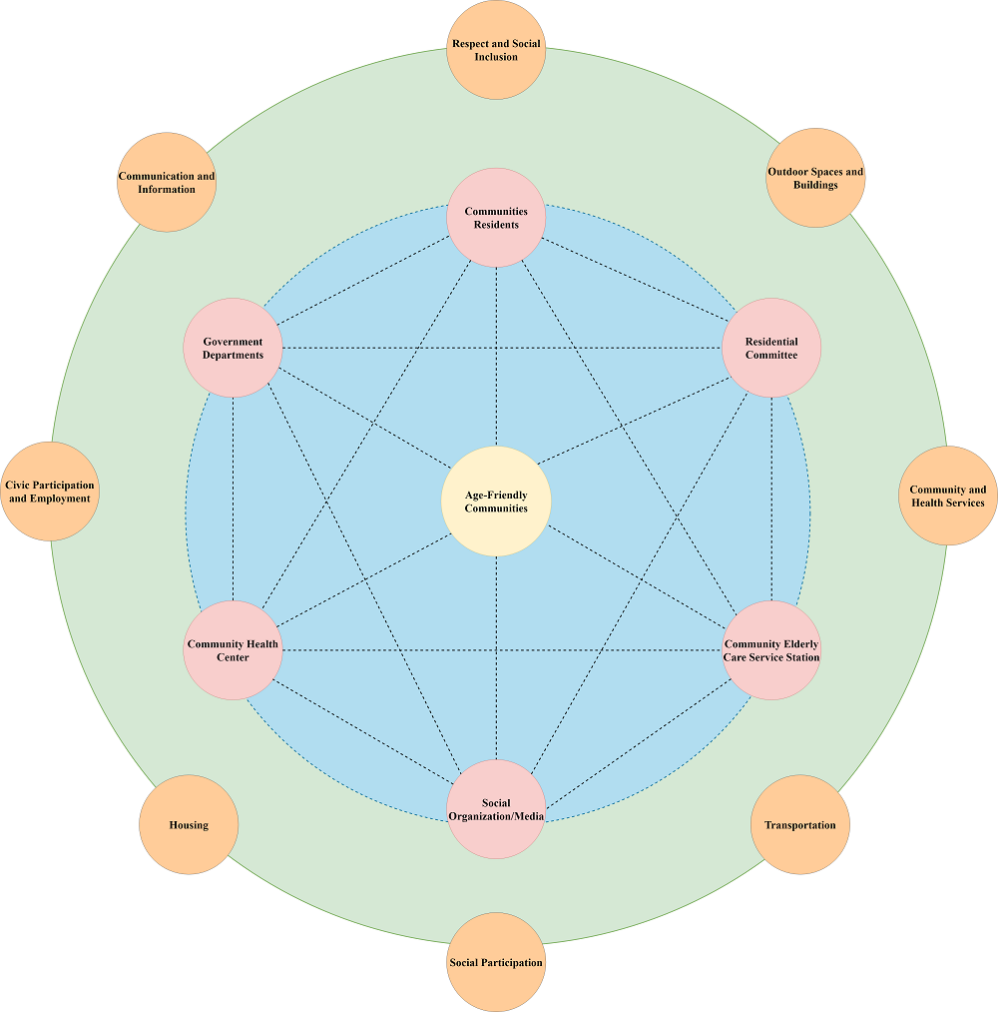
Constructing age-friendly communities as a central goal involves collaboration and participation from multiple stakeholders, including government departments, residential committees, community health centers, community elderly care service stations, social organizations, media, and community residents. Effective cooperation among these stakeholders is necessary to expand the reach of constructing age-friendly communities across all eight domains.

The task of constructing AFCs is complex, dynamic, and multistage, which involves multiple stakeholders’ cooperation. To address the challenges of an aging population and promote AFCIs, collaboration is essential between government departments, residential committees, community health centers, community elderly care service stations, social organizations, media, and community residents (Figure 2).

Government departments should focus on constructing AFCs, by formulating and improving relevant laws and regulations on AFCs, designing scientific and accurate tools for evaluating AFCs, and establishing long-term supervision and feedback mechanisms. Residential committees should first design medium- and long-term plans for promoting AFCIs based on three overarching areas [43]: (1) physical infrastructure (eg, outdoor spaces and buildings, transportation, housing); (2) social opportunities (eg, civic participation, social participation, respect, and social inclusion); and (3) supportive services (eg, communication and information, community and health services). Residential committees should conduct a comprehensive assessment of their respective communities based on AFC policies and tools developed by the government, and identify deficiencies within communities. Our findings show that community residents have urgent needs for old-age care and medical services. Therefore, promoting continuous and integrated community care that combines medical and old-age care is necessary. Additionally, residential committees should explore smart community care services with the help of information technology, treating the community as the basic unit. Besides, it is important to build a smart service platform that connects “Internet+community home care”, with community health centers and elderly care service stations, that can establish a connection between the elderly needs and community service resources. Finally, residential committees should create an atmosphere of age-friendliness within the communities, build more spaces where different aged residents can interact together, and encourage more older adults to participate in the construction of AFCs. Social organizations should adopt age-friendly practices or provide more activities catering to the needs of older adults. Social media should strengthen public awareness of AFCs, ensuring more residents understand the concept of AFCs and their benefits and actively participate in the construction of AFCs. Community residents should also actively cooperate with the work of the communities, improve their cognition of AFCs, and make suggestions for the construction of AFCs.

### Limitations

The findings generated from this study should be interpreted considering several limitations. First, our survey included healthy and cognitively intact older adults, while those who were suffering from disabilities or dementia were excluded. However, understanding the living experience of this excluded group would be vital to help construct AFCs. Second, the representativeness of our study was limited, since we surveyed only a sample from 4 out of 16 districts in Beijing. Future studies should consider conducting similar studies in smaller cities across China and including more related subjects. Finally, our study followed a voluntary participation principle when conducting the questionnaire survey. Female respondents showed more willingness to participate in the survey, resulting in the gender ratio of female respondents (over 70%). Additionally, many surveyed residents never left their living communities, and they did not have a deep understanding of other communities. Future work should further explore the influence of gender, education level, and length of residence on community residents’ perceptions of AFCs.

### Conclusions

China is in the early stages of becoming an age-friendly society. Findings from this study show that community residents in Beijing have a low level of cognition of AFCs, and face many barriers from the perspective of hardware facilities and software facilities. Therefore, it is imperative to take measures to (1) increase age-friendly awareness and sense of belonging among community residents; (2) establish an age-friendly atmosphere and improve communities’ software facilities; (3) improve the living environment and strengthen communities’ hardware facilities; (4) increase the active participation of older persons in the community to enhance their happiness; and (5) and promote AFCIs through joint efforts of multiple stakeholders, including government departments, residential committees, community health centers, community elderly care service stations, social organizations, media, and community residents. Our study provides suggestions for tangible central government policy and practice initiatives and resource allocation. These outcomes have a direct and positive impact on the well‐being of older adults.

## Abbreviations

AFC: Age-Friendly Communities
AFCI: Age-Friendly Communities Initiatives
WHO: World Health Organization

## References

[1] (2024). World population prospects 2024: summary of results. United Nations.

[2] Stenholm S, Westerlund H, Salo P (2014). Age-related trajectories of physical functioning in work and retirement: the role of sociodemographic factors, lifestyle and disease. J Epidemiol Community Health.

[3] Hong A, Welch-Stockton J, Kim JY, Canham SL, Greer V, Sorweid M (2023). Age-friendly community interventions for health and social outcomes: a scoping review. Int J Environ Res Public Health.

[4] Fang ML, Sixsmith J, Hamilton-Pryde A (2022). Co-creating inclusive spaces and places: towards an intergenerational and age-friendly living ecosystem. Front Public Health.

[5] Lawton MP, Nahemow L (1973). The Psychology of Adult Development and Aging.

[6] Menec VH, Means R, Keating N, Parkhurst G, Eales J (2011). Conceptualizing age-friendly communities. Can J Aging.

[7] (2007). Global age-friendly cities: a guide. World Health Organization.

[8] Lai S, Zhou Y, Yuan Y (2021). Associations between community cohesion and subjective wellbeing of the elderly in Guangzhou, China-a cross-sectional study based on the structural equation model. Int J Environ Res Public Health.

[9] Kim K, Buckley TD, Burnette D, Huang J, Kim S (2022). Age-friendly communities and older adults’ health in the United States. Int J Environ Res Public Health.

[10] Hsu HC (2020). Associations of city-level active aging and age friendliness with well-being among older adults aged 55 and over in Taiwan. Int J Environ Res Public Health.

[11] Jeste DV, Blazer DG, Buckwalter KC (2016). Age-friendly communities initiative: public health approach to promoting successful aging. Am J Geriatr Psychiatry.

[12] (2023). National programmes for age-friendly cities and communities: a guide. World Health Organization.

[13] Lui CW, Everingham JA, Warburton J, Cuthill M, Bartlett H (2009). What makes a community age-friendly: a review of international literature. Australas J Ageing.

[14] Dikken J, van den Hoven RFM, van Staalduinen WH, Hulsebosch-Janssen LMT, van Hoof J (2020). How older people experience the age-friendliness of their city: development of the age-friendly cities and communities questionnaire. Int J Environ Res Public Health.

[15] (2015). Measuring the age-friendliness of cities: a guide to using core indicators. World Health Organization.

[16] Meeks S (2022). Age-friendly communities: introduction to the special issue. Gerontologist.

[17] Ide K, Jeong S, Tsuji T (2022). Suggesting indicators of age-friendly city: social participation and happiness, an ecological study from the JAGES. Int J Environ Res Public Health.

[18] (2007). Checklist of essential features of age-friendly cities. World Health Organization.

[19] Huang JY, Hsu HC, Hsiao YL (2022). Developing indicators of age-friendliness in Taiwanese communities through a modified Delphi method. Int J Environ Res Public Health.

[20] Yu R, Wong M, Woo J (2019). Perceptions of neighborhood environment, sense of community, and self-rated health: an age-friendly city project in Hong Kong. J Urban Health.

[21] Wood GER, Pykett J, Daw P (2022). The role of urban environments in promoting active and healthy aging: a systematic scoping review of citizen science approaches. J Urban Health.

[22] Kim K, Buckley T, Burnette D, Kim S, Cho S (2022). Measurement indicators of age-friendly communities: findings from the AARP age-friendly community survey. Gerontologist.

[23] van Hoof J, Marston HR, Kazak JK, Buffel T (2021). Ten questions concerning age-friendly cities and communities and the built environment. Build Environ.

[24] Jiravanichkul S, Pinich S, Sreshthaputra A, Jarutach T (2024). The development of a well-being environment and age-friendly communities assessment criteria using the analytic hierarchy process: a case of Thailand. NJEDP.

[25] (2024). Beijing municipal bureau of statistics [Chinese]. Beijing Statistical YearBook.

[26] Buckner S, Mattocks C, Rimmer M, Lafortune L (2018). An evaluation tool for age-friendly and dementia friendly communities. Work Older People.

[27] Loos E, Sourbati M, Behrendt F (2020). The role of mobility digital ecosystems for age-friendly urban public transport: a narrative literature review. Int J Environ Res Public Health.

[28] Molina-Martínez MÁ, Marsillas S, Sánchez-Román M, Del Barrio E (2022). Friendly residential environments and subjective well-being in older people with and without help needs. Int J Environ Res Public Health.

[29] Wu D, Wang Y, Lam KF, Hesketh T (2014). Health system reforms, violence against doctors and job satisfaction in the medical profession: a cross-sectional survey in Zhejiang Province, Eastern China. BMJ Open.

[30] Zhang T, Wang X (2021). Association of continuity of general practitioner care with utilisation of general practitioner and specialist services in China: a mixed-method study. Healthcare (Basel).

[31] Plouffe L, Kalache A (2010). Towards global age-friendly cities: determining urban features that promote active aging. J Urban Health.

[32] Nemoto Y, Nonaka K, Kuraoka M (2022). Effects of intergenerational contact on social capital in community-dwelling adults aged 25-84 years: a non-randomized community-based intervention. BMC Public Health.

[33] Choi YJ (2022). Understanding aging in place: home and community features, perceived age-friendliness of community, and intention toward aging in place. Gerontologist.

[34] Chaudhury H, Oswald F (2019). Advancing understanding of person-environment interaction in later life: one step further. J Aging Stud.

[35] Lai S, Lu L, Zhou Z (2021). The effects of family physician-contracted service on health-related quality of life and equity in health in China. Int J Equity Health.

[36] Menec VH, Nowicki S (2014). Examining the relationship between communities’ “age-friendliness” and life satisfaction and self-perceived health in rural Manitoba, Canada. Rural Remote Health.

[37] Gao J, Fan C, Chen B (2022). Telemedicine Is becoming an increasingly popular way to resolve the unequal distribution of healthcare resources: evidence from China. Front Public Health.

[38] Jiang J, Wang P (2018). Health status in a transitional society: urban-rural disparities from a dynamic perspective in China. Popul Health Metr.

[39] Ismail M (2020). Regional disparities in the distribution of Sudan’s health resources. East Mediterr Health J.

[40] Rémillard-Boilard S, Buffel T, Phillipson C (2020). Developing age-friendly cities and communities: eleven case studies from around the world. Int J Environ Res Public Health.

[41] Cheng Y, Chen ZL, Wei Y, Gu N, Tang SL (2024). Examining dynamic developmental trends: the interrelationship between age-friendly environments and healthy aging in the Chinese population-evidence from China Health and Retirement Longitudinal Study, 2011-2018. BMC Geriatr.

[42] Gudlavalleti MVS, John N, Allagh K (2014). Access to health care and employment status of people with disabilities in South India, the SIDE (South India Disability Evidence) study. BMC Public Health.

[43] Scharlach AE (2017). Aging in context: individual and environmental pathways to aging-friendly communities-the 2015 Matthew A. Pollack award lecture. Gerontologist.

